# Relationships between functional and structural corticospinal tract integrity and walking post stroke

**DOI:** 10.1016/j.clinph.2012.04.026

**Published:** 2012-12

**Authors:** Gowri Jayaram, Charlotte J. Stagg, Patrick Esser, Udo Kischka, James Stinear, Heidi Johansen-Berg

**Affiliations:** a Department of Biomedical Engineering, Johns Hopkins University School of Medicine, 720 Rutland Avenue, Baltimore, MD 21205, USA; b Centre for Functional Magnetic Resonance Imaging of the Brain (FMRIB), Nuffield Department of Clinical Neurosciences, University of Oxford, Headington, Oxford, UK; c Movement Science Group, School of Life Sciences, Oxford Brookes University, Oxford, UK; d Oxford Centre for Enablement, Headington, Oxford, UK; e Department of Sports and Exercise Science, University of Auckland, Auckland, New Zealand; f Department of Physical Medicine and Rehabilitation, Northwestern University Feinberg School of Medicine, 303 East Chicago Avenue, Chicago, IL, USA

**Keywords:** CST, corticospinal tract, DTI, diffusion tensor imaging, FA, fractional anisotropy, FCR, functional connectivity ratio, M1, primary motor cortex, TMS, transcranial magnetic stimulation, VL, vastus lateralis, Stroke, Locomotion, Motor recovery, TMS, DTI

## Abstract

**Objective:**

Studies on upper limb recovery following stroke have highlighted the importance of the structural and functional integrity of the corticospinal tract (CST) in determining clinical outcomes. However, such relationships have not been fully explored for the lower limb. We aimed to test whether variation in walking impairment was associated with variation in the structural or functional integrity of the CST.

**Methods:**

Transcranial magnetic stimulation was used to stimulate each motor cortex while EMG recordings were taken from the vastus lateralis (VL) bilaterally; these EMG measures were used to calculate both ipsilateral and contralateral recruitment curves for each lower limb. The slope of these recruitment curves was used to examine the strength of functional connectivity from the motor cortex in each hemisphere to the lower limbs in chronic stroke patients and to calculate a ratio between ipsilateral and contralateral outputs referred to as the functional connectivity ratio (FCR). The structural integrity of the CST was assessed using diffusion tensor MRI to measure the asymmetry in fractional anisotropy (FA) of the internal capsule. Lower limb impairment and walking speed were also measured.

**Results:**

The FCR for the paretic leg correlated with walking impairment, such that greater relative ipsilateral connectivity was associated with slower walking speeds. Asymmetrical FA values, reflecting reduced structural integrity of the lesioned CST, were associated with greater walking impairment. FCR and FA asymmetry were strongly positively correlated with each other.

**Conclusions:**

Patients with relatively greater ipsilateral connectivity between the contralesional motor cortex and the paretic lower limb were more behaviorally impaired and had more structural damage to their ipsilesional hemisphere CST.

**Significance:**

Measures of structural and functional damage may be useful in the selection of therapeutic strategies, allowing for more tailored and potentially more beneficial treatments.

## Highlights

► We investigated the relationship between walking impairment after stroke and integrity of the corticospinal tract (CST). ► We used transcranial magnetic stimulation and diffusion tensor imaging to assess CST integrity. ► We demonstrate that patients with more ipsilateral connectivity between the unlesioned M1 and the affected leg had more structural damage to their CST.

## 1 Introduction

Stroke rehabilitation therapies may be more effective if they are tailored to an individual patient’s surviving anatomical and physiological substrates. However, development of such strategies first requires identification of functional and structural measures that are associated with clinical status and that could in future be tested as predictors of outcomes.

Studies of upper-limb hemiparesis suggest that recovery depends in part on the degree of corticospinal tract (CST) damage (Binkofski et al., 1996; Shelton and Reding, 2001; Stinear et al., 2007). In addition, recovery is related to cortical excitability in the contralesional hemisphere, whereby higher levels of excitability and greater ipsilateral activity during paretic hand movement are associated with a poorer outcome (Caramia et al., 2000; Ward et al., 2003, 2006). While a considerable amount of data is available regarding upper limb motor recovery, there are fewer studies of structural and functional correlates of recovery of the lower limb.

Fundamental differences in the neural control of unilateral hand movements and more automated, bilateral movements of the lower limb such as walking, disqualify conclusions from upper limb studies being directly applied to the lower limb (Luft et al., 2002). In particular, the relationship between the degree of damage to the corticospinal tract and walking impairment remains unclear (Ahn et al., 2006; Dawes et al., 2008). There is some evidence of increased activity in the ipsilateral (contralesional) motor cortex during paretic lower limb movements in more severely impaired patients (Enzinger et al., 2009, 2008; Jang et al., 2005; Luft et al., 2005) but the functional and clinical significance of such activity is unclear.

We used transcranial magnetic stimulation (TMS) to directly assess functional connectivity from the motor cortex of each hemisphere to both lower limbs in chronic stroke patients with persistent paresis of their lower limb. We used diffusion tensor imaging (DTI) to assess the structural integrity of the CST in each hemisphere. We hypothesized that patients with a higher degree of structural damage to the CST in the lesioned hemisphere would have greater relative functional connectivity from the contralesional motor cortex to the ipsilateral paretic limb and greater walking impairment.

## 2 Methods

Nine individuals with persistent hemiparesis due to chronic stroke (⩾6 months) were recruited (see Table 1). All patients provided written informed consent in accordance with local ethical approval and the 2008 Declaration of Helsinki. Patients were screened for contradictions to MRI and TMS and any other cause for their reduced motor function. Patients participated in one clinical/neurophysiology data collection session and one Magnetic Resonance Imaging (MRI) session where DTI and structural imaging was acquired.

### 2.1 Clinical and neurophysiological testing

Each participant’s overall impairment was assessed using the lower-limb section of the Fugl-Meyer (FM) scale (Fugl-Meyer et al., 1975), with higher scores reflecting greater function (maximum 34). Walking impairment measures were derived using a 10 m timed walk (Supplementary information, methods).

TMS was used to stimulate each motor cortex while EMG recordings were taken from the vastus lateralis (VL) bilaterally; these EMG responses were used to calculate both ipsilateral and contralateral recruitment curves for each lower limb (Supplementary information, methods). TMS motor evoked potentials (MEPs) were elicited every 5 s. The slope of each curve was obtained by fitting a line to each subject’s data. All fits had *r* > 0.85. To quantify the balance between ipsilateral and contralateral connectivity, a functional connectivity ratio was calculated for each leg separately:

$$\mathrm{Functional}\;\mathrm{Connectivity}\;\mathrm{Ratio}\;(\mathrm{FCR})=\frac{\mathrm{Slope}\;\mathrm{of}\;\mathrm{Ipsilateral}\;\mathrm{Recruitment}\;\mathrm{Curve}}{\mathrm{Slope}\;\mathrm{of}\;\mathrm{Contralateral}\;\mathrm{Recruitment}\;\mathrm{Curve}}$$

Because of the close proximity of the two lower limb motor cortices and the low spatial resolution of TMS, it is assumed that all responses were a mix of ipsilateral and contralateral inputs to motor neurons but that inputs from the stimulated hemisphere would predominate each measurement, as we have shown previously (Madhavan et al., 2010) FCR values of >1.0 were therefore interpreted as reflecting predominantly ipsilateral connectivity between motor cortex and lower limb motor neurons and FCR values of <1.0 were interpreted as reflecting predominantly contralateral connectivity (Madhavan et al., 2010).

### 2.2 MRI data acquisition

In order to assess the structural integrity of the corticospinal tracts, we acquired diffusion-weighted (three acquisitions of 60 directions, *b* value = 1000 s/mm^2^, voxel dimensions = 2 × 2 × 2 mm, 60 slices, TR = 8.9 s, TE = 93 ms) and T1-weighted data (three acquisitions, voxel dimensions = 1 × 1 × 1 mm, FOV = 256 × 265 mm, TR = 12 s, TE = 5.65 ms) using a Siemens Avanto 1.5T MRI system. Due to technical issues of subject/scanner compatibility, two patients were scanned on a 3T Siemens Trio using identical parameters and four patients were scanned on a 3T Verio using identical parameters.

### 2.3 MRI data analysis

Image analysis was conducted with the Oxford Center for Functional MRI of the Brain (FMRIB)’s Software Library (http://www.fmrib.ox.ac.uk/fsl) (Smith et al., 2004).

Diffusion weighted imaging (DWI) data were analyzed using FMRIB’s Diffusion Toolbox (Supplementary information). Estimated parameters included fractional anisotropy (FA), a scalar ranging from 0 to 1, which quantifies the directional dependence of the diffusion signal and has been used as a measure of white matter integrity.

#### 2.3.1 Anatomically-defined ROI analysis

The posterior limb of the internal capsule (PLIC) was manually delineated bilaterally from the level of the anterior commissure to the base of the corona radiata in standard space. Mean FA was computed for both the lesioned and non-lesioned PLICs and used to calculate the asymmetry of PLIC FA. To ensure that any correlations found were specific to the PLIC and not a reflection of a more global effect, the FA asymmetry of a control ROI in the anterior limb of the internal capsule (ALIC) was also calculated for each patient in an identical manner. Using this calculation method symmetrical FA values yield an FA asymmetry value of 0 and greater inter-hemispheric asymmetry in the PLIC FA values yield a value closer to 1:

$$\mathrm{FA}\;\mathrm{Asymmetry}=\frac{\mathrm{FA}\;\mathrm{unaffected}-\mathrm{FA}\;\mathrm{affected}}{\mathrm{FA}\;\mathrm{unaffected}+\mathrm{FA}\;\mathrm{affected}}$$

#### 2.3.2 Whole brain analysis

Voxel-wise correlations between FA and the Functional Connectivity Ratio (FCR) as assessed by TMS were calculated using Tract Based Spatial Statistics (TBSS) (Supplementary information).

#### 2.3.3 Lesion volume and overlap with reconstructed corticospinal tract

In order to investigate whether the degree to which a stroke interrupts the CST correlates with FCR and whether it could be considered a marker for the level of impairment, we also calculated the overlap between stroke lesions and probabilistic maps of the corticospinal tract derived from DTI (Supplementary information).

### 2.4 Statistical analysis

SPSS software (SPSS Inc., Chicago, USA), using two-tailed Spearman’s Rho non-parametric test, was used to evaluate bivariate correlations between TMS, MRI and behavioral measures. Simple and multiple linear regressions were performed to determine the strongest marker of FCR and of behavioral impairment. A repeated measures ANOVA including within subject factors of leg (paretic, non-paretic) and side of stimulation (contralateral, ipsilateral), along with follow up two-tailed paired samples *t*-test were used to test for differences in the slopes of ipsilateral and contralateral recruitment curves. The adopted level of significance was set at 0.05. All *p* values have been corrected for multiple comparisons. Therefore, any *p* value greater than one has been represented as one.

## 3 Results

We studied 13 patients with a mean FM score of 23.8 (range 12–34), and a mean walking speed of 39.8 m/min (range 5.4–89.7) (Table 1).

### 3.1 Assessing balance between contralateral and ipsilateral functional connectivity with TMS

**Fig. 1:**
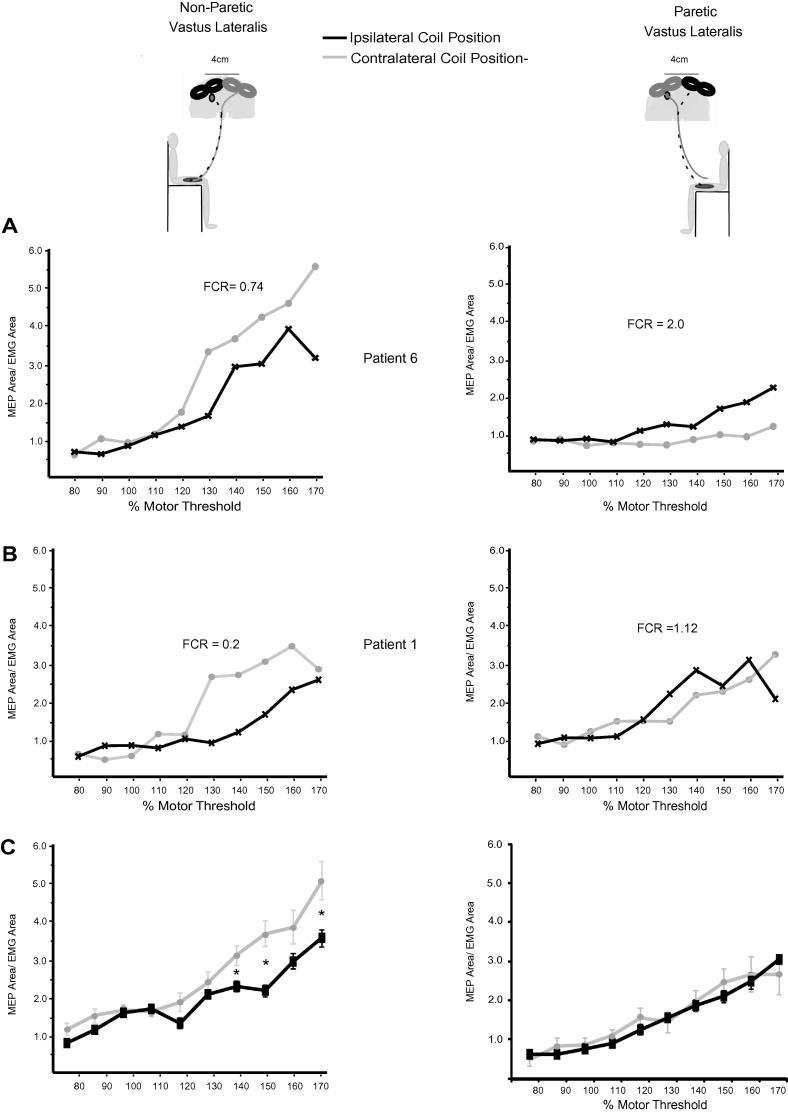
Bilateral TMS recruitment curves for two representative patients (A and B) and the group (C). The left hand column shows recruitment curves for the non-paretic VL, the right hand column from the paretic VL. For the non-paretic VL, the contralateral coil position (grey line) elicits a steeper recruitment curve than the ipsilateral position (black line) whereas the opposite is true for the paretic VL, resulting in a significant interaction between leg and side of stimulation. Each data point is calculated as the mean of the MEP area divided by pre-trigger EMG area for each block and is plotted as a function of increasing TMS intensities normalized to motor threshold of the respective leg and coil position.

We demonstrated a difference in the relative amount of ipsilateral and contralateral conductivity for the paretic versus the non-paretic leg (Fig. 1). A repeated measures ANOVA of recruitment curve slopes revealed a significant interaction between leg and side of stimulation (*F*(1,12)=7.5, *p* = 0.026). For the non-paretic VL, the recruitment curve slope is significantly steeper for stimulation of the contralateral (contralesional) hemisphere than for stimulation of the ipsilateral (lesional) hemisphere (*t* = 2.63, *df* = 12, corrected *p* = 0.03). Motor thresholds for all four stimulation configurations are reported in Supplementary Table S1.

For each subject and each leg we quantified the relative difference in slopes for stimulation of the ipsilateral versus contralateral hemisphere by calculating a functional connectivity ratio (FCR). The group mean FCR of the non-paretic VL was 0.55 (range 0.2–0.83) consistent with contralateral motor control. For the paretic VL, there was greater variability in the relationship between the ipsilateral and contralateral recruitment curves (e.g., compare Fig. 1A and B); group mean FCR was 1.7 (range 0.27–2.6), suggestive of predominantly ipsilateral motor control. Across the group, FCR values for the paretic limb were significantly higher than those for the non-paretic limb (*t* = 4.8, *df* = 12, *p* < 0.001) reinforcing the differences in the balance between contralateral and ipsilateral control of the two limbs (for group data see Fig. 1C).

**Fig. 2:**
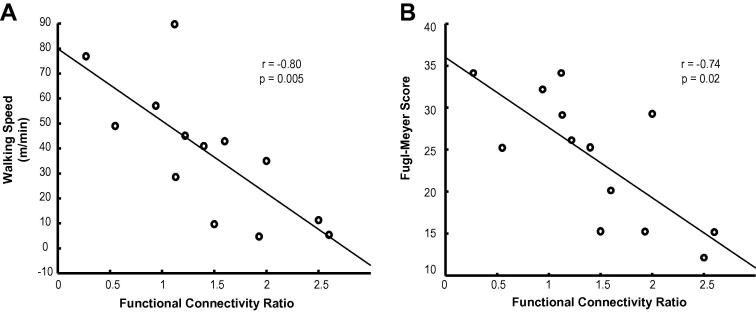
Correlations between FCR and clinical outcome measures. (A) The relationship between FCR of the paretic VL and walking speed from a 10 m timed walk. Increasing values of ipsilateral connectivity are associated with slower walking speeds. (B) The relationship between FCR of the paretic VL and lower limb Fugl-Meyer score (max 34). Increasing values of ipsilateral connectivity are associated with lower FM scores and greater disability.

The FCR of the paretic VL was negatively correlated with walking speed (*r* = −0.80, corrected *p* = 0.005) and lower limb Fugl-Meyer score (*r* = −0.74, corrected *p* = 0.02) (Fig. 2), such that patients with greater relative ipsilateral connectivity to the paretic leg had a slower walking speed and a poorer clinical outcome. The FCR of the non-paretic VL was not related to walking speed (*r* = −0.06, corrected *p* = 1) or to Fugl-Meyer score (*r* < −0.01, corrected *p* = 1) (data not shown).

### 3.2 Assessing relationship between functional and structural measures of connectivity

#### 3.2.1 Region of interest analyses

**Fig. 3:**
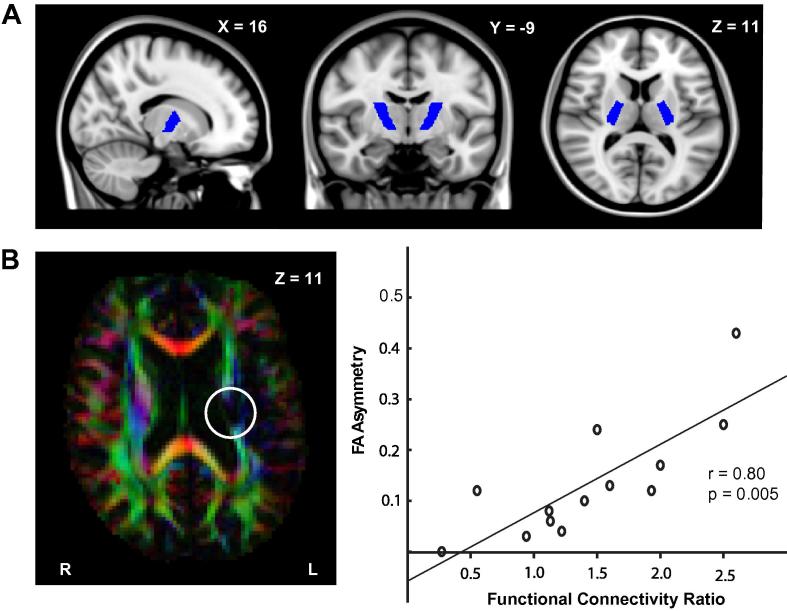
Correlations between FA asymmetry and FCR. (A) Representative slices showing the posterior limb of the internal capsule (PLIC) masks used as a region of interest for ROI based FA calculations bilaterally. FA asymmetry was calculated using the mean FA values within these PLIC ROIs. (B) A representative axial slice from a composite map of FA and the principal diffusion direction from patient 5 (FA asymmetry = 0.24), shown in RGB (Red = Left–Right; Green = Anterior–Posterior, Blue = Superior–Inferior). This illustrates the diminished FA within the descending PLIC in the lesioned hemisphere (white circle). (C) The relationship between FA asymmetry of the PLIC and FCR. Increasing values of FA asymmetry are associated with increasing ipsilateral connectivity.

There was a significant decrease in FA within the ipsilesional PLIC (mean = 0.42 ± 0.02) when compared with the contralesional PLIC (mean = 0.54 ± 0.01) (*t* = 5.07, *df* = 12, corrected *p* < 0.005) (Table 1; Fig. 3B).

There was a strong positive relationship between the TMS-derived FCR measure and FA asymmetry within the PLIC (Fig. 3C). Patients with greater FA asymmetry had a higher FCR for their paretic lower limb (*r* = 0.80, corrected *p* = 0.005), reflecting greater reliance on ipsilateral connections (from the contralesional hemisphere) compared to contralateral connections (from the ipsilesional hemisphere). FA asymmetry did not correlate with the FCR of the non-paretic limb (*r* = −0.55, corrected *p* = 1). There was no relationship between the FA asymmetry of the anterior limb of the internal capsule, our control ROI, and FCR (*r* = 0.09, corrected *p* = 1; data not shown).

FA asymmetry was negatively correlated with FM score (*r* = −0.78, corrected *p* = 0.005) and tended to be negatively correlated with walking speed (*r* = −0.68, corrected *p* = 0.05) such that patients with a greater asymmetry showed slower walking and had greater functional impairment.

When the four patients scanned at 3T were removed from the analysis, all significant relationships were maintained.

#### 3.2.2 Whole brain analysis

TBSS was used to test across the group for correlations between FCR and voxel-wise measures of FA. Negative correlations between FA and FCR were found in multiple white matter pathways of the lesioned hemisphere, including parts of the CST, suggesting that greater damage to these regions of the lesioned hemisphere was associated with greater relative reliance on ipsilateral functional connectivity from the contralesional hemisphere to the paretic leg (Supplementary information, results). A positive correlation between FA and FCR was found within the PLIC of the non-lesioned hemisphere (Supplementary information, results).

No significant relationships were found between FCR or impairment and lesion volume or lesion overlaps with the CST (Supplementary information, results). A step-wise multiple regression found that FCR of the paretic leg was the strongest marker of functional impairment (Supplementary information, results).

## 4 Discussion

This study investigated the inter-relationship between the structural integrity and functional connectivity of the CST and the extent of lower limb motor impairment in chronic stroke patients. Patients with relatively greater ipsilateral connectivity between the contralesional motor cortex and the paretic lower limb were more behaviorally impaired and had more structural damage to their ipsilesional hemisphere CST.

This is the first report, to our knowledge, that utilises TMS to examine ipsilateral and contralateral cortical functional connectivity with proximal lower limb muscle motor neurons of the paretic limb following stroke. The increased ipsilateral conductivity to the lower limbs found here is consistent with previously reports of increased ipsilateral control of upper limbs after cerebral damage (Alagona et al., 2001; Hendricks et al., 1997; Netz et al., 1997; Trompetto et al., 2000; Turton et al., 1996).

In addition to a correlation between functional CST connectivity and behavioral outcomes, we also demonstrated a relationship between functional CST connectivity and structural integrity of the CST. For the paretic leg, the functional connectivity ratio (FCR), was strongly correlated with the FA asymmetry of the PLIC (Fig. 3) and a whole-brain analysis confirmed that increased FCR for the paretic leg was associated with multiple clusters of reduced FA in the ipsilesional hemisphere, many of which were located within the CST (Supplementary information). Interestingly, a single cluster of positive correlation in the PLIC of the contralesional hemisphere was found (Supplementary information), where greater FCR for the paretic leg was associated with increased FA. Together, these results suggest that greater relative ipsilateral functional connectivity to the paretic limb may arise predominantly from loss of structural integrity of the ipsilesional (contralateral) CST, but also depends to some degree on the integrity of white matter in the contralesional (ipsilateral) CST. Whether these relationships reflect pre-existing variation in white matter structure (Johansen-Berg et al., 2007; Tomassini et al., 2011), or degenerative or compensatory changes secondary to damage (Crofts et al., 2011; Schaechter et al., 2009) cannot be addressed by the current study.

The physiological and imaging measures tested here have clinical relevance, as shown by correlations with impairment. While FA showed some associations with walking impairment, FCR was found to be a stronger marker. This finding complements a previous report showing FA asymmetry was only related to Fugl-Meyer score in more behaviorally impaired patients, (Stinear et al., 2007). However, the potential of these functional and structural measures to predict *future* walking recovery remains to be tested.

We found no correlations between lesion volume (or lesion overlap with CST) and impairment or functional or structural connectivity values (Supplementary information). Therefore, it appears that simple measures of lesion volume do not indicate the level of impairment or functional CST connectivity and that FA asymmetry provides a more relevant measure of the structural integrity of CST than does lesion overlap with CST.

Our finding of relationships between impairment and FCR or FA asymmetry raises the possibility that such measures could be used to tailor individual therapeutic interventions, such as brain stimulation approaches (Hummel et al., 2005; Mansur et al., 2005). For example, in a patient with little residual integrity of the ipsilesional CST and strong ipsilateral control of the paretic limb, facilitatory stimulation of the lesioned hemisphere is likely to have little beneficial effect and facilitatory stimulation might be best applied over the contralesional hemisphere to enhance its effectiveness in controlling lower limb movement.

We recognize certain limitations to our study. We did not study healthy control subjects and so cannot say whether the relationships detected are specific to the stroke population. Rather, we focused on characterizing clinical heterogeneity within a patient group, with the aim of informing future attempts for individualizing therapies. Further, we studied a relatively small number of patients with heterogenous stroke volumes, locations and etiology. Although similar group sizes have been used in a number of prior imaging or TMS studies of stroke (Stinear et al., 2007; Ward et al., 2006) larger studies should be carried out in future to test these effects across the wider stroke population and to allow for subgroup comparisons to determine any influence of patient heterogeneity.

The relationships between functional and structural measures of the corticospinal tract and behavioral outcome presented here further our understanding of the factors that may influence walking recovery post stroke and demonstrate the complimentary nature of neurophysiological and imaging techniques in characterizing a patient’s residual anatomical and physiological substrates. In the future, such measures may inform the selection of therapeutic strategies, moving towards more individualized treatments that will optimize a patient’s potential for recovery.

## Figures and Tables

**Table 1:** Clinical Details.

Subject	Age/sex	Time post stroke (years)	Stroke hemisphere	Lesion location	Mobility aid	FM	Walking speed (m/min)	FA asymmetry	Affected limb FCR	Lesion volume (mm^3^)	Lesion overlap (mm^3^)
1	60/M	4.5	L	Frontal lobe	None	34	89.75	0.08	1.11	148,248	320
2	56/M	4.7	L	Intracerebral hemorrhage	AFO	25	40.95	0.1	1.42	2016	0
3	64/M	1.8	R	Subcortical infarct	Stick	26	45.11	0.04	1.21	608	392
4	74/F	1.8	L	MCA territory	AFO/tripod	15	4.75	0.12	1.96	3752	176
5	77/F	2.8	L	MCA territory	AFO	15	9.68	0.24	1.55	2432	112
6	65/M	1.3	R	MCA territory	None	29	35	0.17	2.05	2392	376
7	73/M	2.7	R	MCA/PCA territory	AFO/stick	15	5.4	0.43	2.63	355,960	440
8	79/M	2.0	L	Caudate	None	34	76.9	0	0.27	727	232
9	70/M	1.0	R	Frontal lobe	AFO/stick	20	42.9	0.13	1.62	6496	0
10	66/M	4.6	R	Occipital temporo-parietal	Cane	29	25.6	0.06	1.13	45,736	6220
11	56/M	11.3	L	Putamen	None	32	57.1	0.03	0.94	108,630	1308
12	57/F	4.0	R	Parietal lobe	Tripod	12	11.3	0.25	2.5	26,162	235
13	72/M	4.9	R	Preceneous cortex	AFO	25	49	0.12	0.55	22,204	0

AFO – Ankle Foot Orthosis.
