# Supplementary material for: Relationships between functional and structural corticospinal tract integrity and walking post stroke
Source: Clin Neurophysiol. 2012 Dec;123(12):2422–8. doi: 10.1016/j.clinph.2012.04.026 (PMC3778984; doi:10.1016/j.clinph.2012.04.026)
Supplement: Supplementary data 1 — Supplementary material [file mmc1.doc]

*Supplementary Methods*

**Clinical assessment**

Walking impairment was characterized by a measure of walking speed derived from a 10 meter timed walk. For this test, subjects were positioned approximately 1 meter behind the start of the 10 meter course. They were instructed to walk at a comfortable rate until 1 meter past the end of the course. This was done three times and averaged.

**TMS and Electromyography**

EMG recordings were performed using surface disposable neonatal ECG electrodes (Henley’s Medical Ltd, Welwyn, UK) placed bilaterally over the vastus lateralis (VL) in a belly-tendon montage. The ground electrode was placed over the right knee. EMG data were sampled at 2000 Hz, amplified (1000x) and bandpass-filtered (10-1000 Hz) using a CED 1902 isolated pre-amplifier (Cambridge Electronic Design, UK). EMG and stimulator trigger pulse data were recorded using Spike2 software version 7 (Cambridge Electronic Design, UK). A 110 mm double cone coil (Magstim, UK) was placed to preferentially stimulate either the lesioned or non-lesioned M1. We measured the amplitude of VL EMG activity, of individual legs, during 3 maximum voluntary contractions (MVCs) performed against resistance allowing for a brief rest period in between. During all TMS measures patients were instructed to maintain a contraction of the muscle under investigation at 20% of their MVC using visual feedback. Using a custom written Spike2 script TMS pulses were triggered only when the EMG activity was in the target range (20 + 1% of MVC). If subjects were having difficulty maintaining this contraction or reported being fatigued, they were allowed to rest. All data were stored for off-line analysis using a custom written Matlab program (Mathworks, MA, USA).

A double cone coil (Magstim, UK) was placed1 cm posterior to the vertex, 2 cm to the left or to the right to preferentially stimulate either the lesioned or non-lesioned M1. For each coil position, the active motor threshold (aMT) was determined for individual legs, as the lowest intensity of magnetic stimulation required to evoke MEPs of 100μV in amplitude in five out of ten trials. TMS recruitment curves were collected for each leg and each coil position and consisted of randomized blocks of eight MEPs elicited at stimulus intensities between 80% and 170% of aMT in 10% increments. When the coil was positioned over the lesioned M1 and EMG electrodes placed on the paretic limb, we obtained a *contralateral* recruitment curve, as the coil was contralateral to the muscle of interest. Likewise, *ipsilateral* recruitment curves were constructed from MEPs elicited in the paretic leg when the coil was positioned over the non-lesioned M1. Data to calculate contralateral and ipsilateral recruitment curves were collected for both paretic and non-paretic legs. We randomized the order each hemisphere (lesioned or non-lesioned) was stimulated across the group and alternated which leg (paretic and non-paretic) was being assessed to prevent fatigue, since MEPs were elicited with a tonic contraction in the muscle. Therefore, a sample order might be 1. Lesioned hemisphere paretic leg 2. Lesioned hemisphere non-paretic leg 3. Non-lesioned hemisphere paretic leg 4. Non-lesioned hemisphere non-paretic leg. Motor threshold for the four coil configurations are reported in Supplementary Table S1.

Supplementary Table S1. Motor Thresholds (% Maximum Stimulator Output).


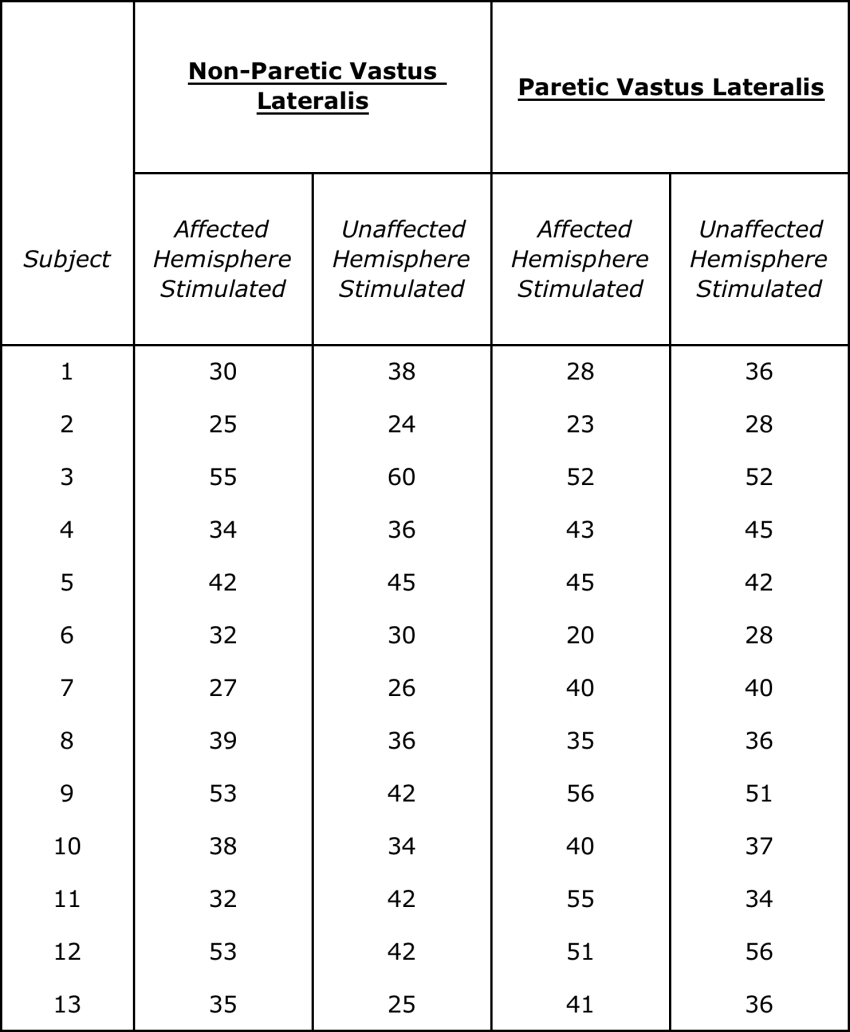


**MRI data analysis**

First raw diffusion data were corrected for motion and eddy current effects. Next, a diffusion tensor model (Basser et al., 1994) was fit to the raw diffusion data at each voxel. This allowed for voxel-wise estimates of diffusion parameters to be calculated. Estimated parameters included fractional anisotropy (FA), a scalar ranging from 0 to 1, which quantifies the directional dependence of the diffusion signal. FA has been used as a measure of white matter integrity as it reflects microstructural properties including axonal architecture, the density of axonal fibres and the extent of myelination (Beaulieu, 2002). All patients’ FA maps were aligned into a common space using FMRIB’s Nonlinear Image Registration Tool (FNIRT) (Klein et al., 2009).

*Tract-based Spatial Statistics (TBSS)*: For voxel-wise analyses to be interpretable, imaging data from patients with left hemispheric strokes were mirrored about the midline so that all lesions were located in the right hemisphere. Then, whole-brain voxel-wise analyses of FA were performed using tract based spatial statistics (TBSS), which is described in detail elsewhere (Smith et al., 2006). Briefly, a mean FA image was created from the non-linearly aligned FA maps and this mean FA image was thinned to create an FA skeleton which represents the centers of all tracts common to the group. Each subject's aligned FA data were then warped onto this skeleton by searching perpendicular from the skeleton for maximal FA values. The resulting aligned, skeleton-projected data were fed into voxelwise cross-subject statistics. A general linear model (GLM) approach was used to positively and negatively correlate FCR values with voxel-wise FA values on the skeleton. Statistical inference was performed using FSL’s Randomise tool to carry out permutation based testing using a one-tailed statistical threshold of t > 3.6 (df = 12; uncorrected p < 0.005). Surviving clusters containing more than 10 voxels are reported and were used as seed masks for probabilistic tractography (as described above) to visualize pathways passing through white matter regions where FA correlated with TMS and clinical measures.

*Lesion volume and overlap with reconstructed corticospinal tract*

In order to investigate whether the degree to which a stroke interrupts the CST correlates with FCR and predicts impairment, we also calculated the overlap between stroke lesions and probabilistic maps of the corticospinal tract derived from DTI. As for the voxel-wise analysis above, imaging data from patients with left hemispheric strokes were mirrored about the midline so that all lesions were located in the right hemisphere. Lesions were manually delineated on individual subject co-registered T1-weighted images in FSLview and were defined as areas of focal hypointensity with a signal intensity between cortex and CSF. The volume of each patient’s lesion was calculated. Lesion maps were then registered into standard space using non-linear registration. We used a probabilistic map of corticospinal tract created previously (Dawes et al., 2008), based on probabilistic tractography using DTI data in eight healthy controls, and thresholded to include only those voxels present in at least two individuals. The overlap (in mm^3^) between the lesion volumes and this probabilistic CST map in standard space was calculated for each individual patient.

Simple and multiple linear regressions were performed to determine the strongest predictors of FCR and of behavioural impairment.

*Supplementary Results*

*Tract-based Spatial Statistics*

TBSS was used to test for voxel-wise correlations between FA and FCR across the whole brain white matter skeleton. FCR values were negatively correlated with FA within clusters in the external capsule, corpus callosum,,middle cerebral peduncle, posterior limb of the internal capsule, uncinate fasciculus and thalamic radiation of the *lesioned hemisphere* (t > 3.6, p < 0.005 ; Supplementary Figure S1, Supplementary Table S2). The negatively correlated cluster in the cerebral peduncle, and posterior limb of the internal capsule of the lesioned hemisphere generated CST paths in the lesioned hemisphere (Supplementary Figure S1). Tractography from the cluster in the corpus callosum also generated corticospinal tracts passing through the corpus callosum and reaching up towards the lower limb motor cortex (Supplementary Figure S1). Interestingly, FCR values were positively correlated with FA within a small cluster in the longitudinal fasciculus of the *non-lesioned hemisphere* (t > 3.6, p < 0.005; Supplementary Figure S1, Supplementary Table S2) that generated CST paths (Supplementary Figure S1).

Supplementary Table S2. Details of clusters showing significant correlations between ICI and FA.


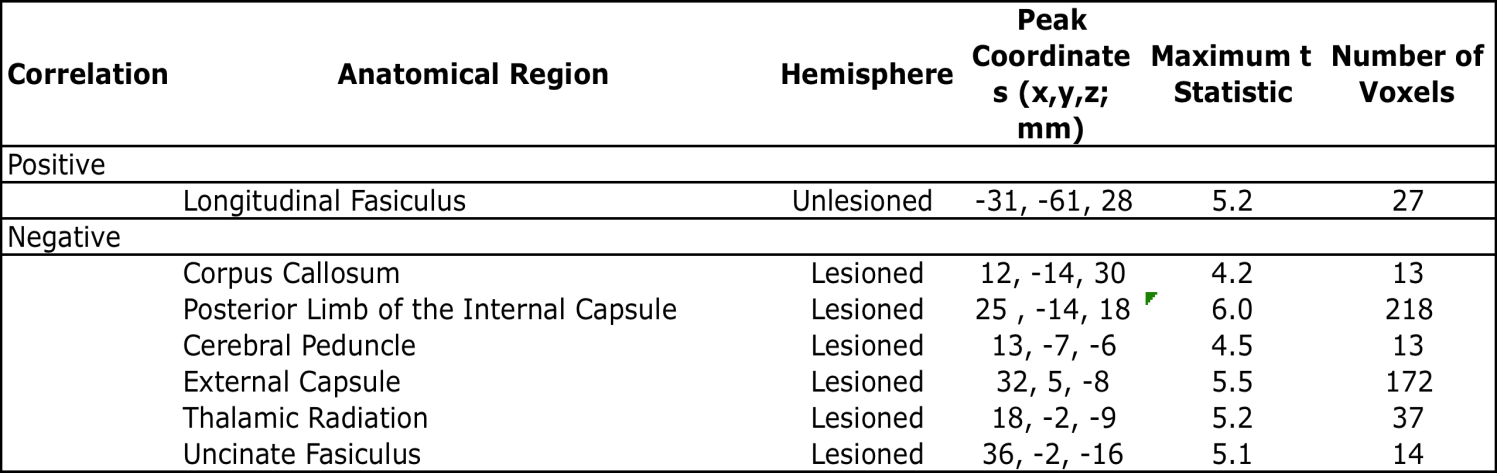


*Lesion volume and overlap with reconstructed corticospinal tract*

Table 1 of the main paper presents the total lesion volume (range 608 – 355960 mm^3^) for all the patients and the total volume of overlap with a reconstructed corticospinal tract (range 0 – 2436 mm^3^). There was no relationship between lesion volume or lesion overlap and FCR values (r = 0.20, r = 0.022 respectively; p > 0.4), walking speed (r = -0.17, r = 0.02; p > 0.6), or Fugl-Meyer score (r = -0.11, r = 0.35; p > 0.2)

**Multiple regression of clinical prediction**

A step-wise multiple regression was performed to evaluate FCR of the paretic VL and FA asymmetry as predictors of clinical measures. Considered alone, FCR of the paretic leg was found to strongly predict walking speed (R = 0.75, F(1,12) = 14.0, corrected P = 0.015) and Fugl Meyer score (R = 0.74, F(1,12) = 13.61, corrected P = 0.02). However, in all cases, the predictive power of the models was reduced when FA asymmetry was also included as a predictor of clinical measures (R = 0.76, F(2,12) = 7.0, corrected P = 0.065 and R = 0.78, F(2,12) = 7.89, corrected P = 0.45 respectively), despite the observation that FA asymmetry alone had some predictive power for the clinical variables (R = 0.69, F(1,12) = 10.08, corrected P = 0.045 and R = 0.74, F(1,12) = 12.8, corrected P = 0.02 respectively). FCR of the paretic leg was therefore found to be a stronger predictor of functional impairment than was FA asymmetry.

**­­­References**

Basser PJ, Mattiello J, LeBihan D. Estimation of the effective self-diffusion tensor from the NMR spin echo. J Magn Reson B 1994;103:247-54.

Beaulieu C. The basis of anisotropic water diffusion in the nervous system - a technical review. NMR Biomed 2002;15:435-55.

Dawes H, Enzinger C, Johansen-Berg H, Bogdanovic M, Guy C, Collett J, et al. Walking performance and its recovery in chronic stroke in relation to extent of lesion overlap with the descending motor tract. Exp Brain Res 2008;186:325-33.

Klein A, Andersson J, Ardekani BA, Ashburner J, Avants B, Chiang MC, et al. Evaluation of 14 nonlinear deformation algorithms applied to human brain MRI registration. Neuroimage 2009;46:786-802.

Smith SM, Jenkinson M, Johansen-Berg H, Rueckert D, Nichols TE, Mackay CE, et al. Tract-based spatial statistics: voxelwise analysis of multi-subject diffusion data. Neuroimage 2006;31:1487-505.
