# Supplementary material for: Relationships between functional and structural corticospinal tract integrity and walking post stroke
Source: Clin Neurophysiol. 2012 Dec;123(12):2422–8. doi: 10.1016/j.clinph.2012.04.026 (PMC3778984; doi:10.1016/j.clinph.2012.04.026)
Supplement: Supplementary data 2 — Supplementary Fig. S1 [file mmc2.doc]

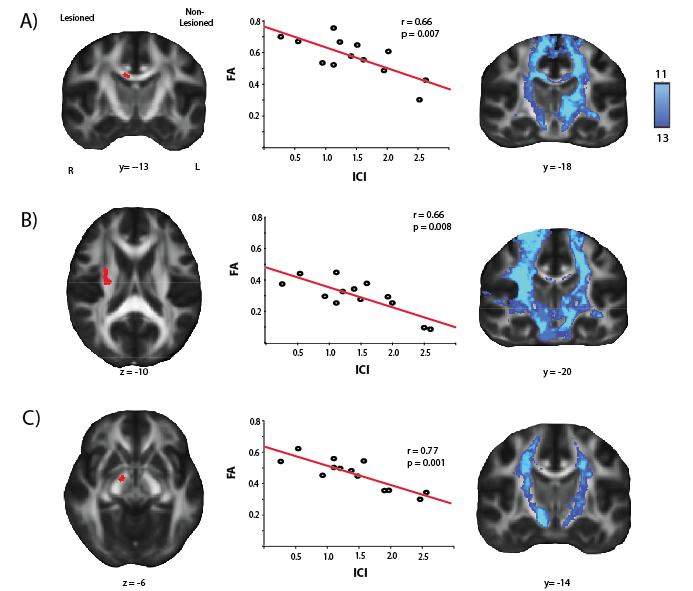


**Supplementary Figure S1.**

Whole-brain correlations between FA and ICI, and probabalistic tractography (PDT) paths from local areas of correlation. (Left) Selected clusters in which the FA is significantly correlated with ICI values (t > 3.6) are overlaid in red on the mean FA image from all subjects. Clusters were dilated by one voxel for ease of visualization. (Center) Scatter plots illustrating the significant correlations between the mean FA within these clusters and ICI values (Right) Group probability maps of tracts were generated by PDT from the respective correlated clusters and overlaid onto the mean FA image from all participants.

A] Cluster in the lesioned corpus callosum *negatively* correlated with individual ICI values (r = 0.66; p = 0.007), and group probability maps of corticospinal tracts passing through the corpus callosum generated by PDT from this cluster.

B] Cluster in the posterior limb of the internal capsule *negatively* correlated with individual ICI values(r = 0.77; p = 0.008), and group probability maps of corticospinal tract generated by PDT from this cluster.

C] Cluster in the lesioned cerebral peduncle *negatively* correlated with individual ICI values(r = 0.77; p = 0.001), and group probability maps of both corticospinal tracts

generated by PDT from this cluster.
